# Temperament in trichotillomania and skin picking disorder

**DOI:** 10.12788/acp.0096

**Published:** 2023-05-01

**Authors:** Jon E. Grant, Stephanie Valle, Ibrahim Aslan, Eve K. Chesivoir, Samuel R. Chamberlain

**Affiliations:** Department of Psychiatry and Behavioral, Neuroscience, University of Chicago Pritzker School of Medicine, Chicago, Illinois, USA; Department of Psychiatry and Behavioral, Neuroscience, University of Chicago Pritzker School of Medicine, Chicago, Illinois, USA; Department of Psychiatry, Southampton University, Southampton, UK; Department of Psychiatry and Behavioral Neuroscience, University of Chicago Pritzker School of Medicine, Chicago, Illinois, USA; Department of Psychiatry, Southampton University, Southampton, UK

## Abstract

**Background:**

Trichotillomania (TTM) and skin picking disorder (SPD) result in significant psychosocial burden. Despite this burden, however, risk factors related to the development of these disorders remain unclear. The present study assessed temperament in a well-characterized sample of adults with TTM or SPD.

**Methods:**

A total of 202 adults age 18 to 65 were enrolled; 44 had TTM, 30 had SPD, and 128 served as controls. Participants completed the self-report Tridimensional Personality Questionnaire (TPQ) to examine the severity of TTM and SPD symptoms, quality of life, and temperament. Group differences were characterized and correlations with other measures were examined.

**Results:**

Compared to controls, those with TTM or SPD scored significantly higher on harm avoidance and its subscales, with TTM associated with higher scores than SPD. Those with TTM or SPD scored significantly higher on only 1 measure of novelty seeking (extravagance). Higher TPQ harm avoidance correlated with worse hair pulling severity and worse quality of life.

**Conclusions:**

The temperament traits of participants with TTM or SPD differed in significant ways from controls; those with TTM or SPD generally demonstrated similar trait profiles. A dimensional approach to the personalities of those with TTM or SPD may offer insight and provide clues to treatment strategies.

## Introduction

Trichotillomania (TTM), characterized by repetitive hair pulling, and skin picking disorder (SPD), defined by repetitive skin picking that results in lesions, are psychiatric disorders that may result in significant psychosocial impairment, reduced self-esteem, and even medical complications.^1^ Factors that may contribute to the development of TTM or SPD, however, are still largely unknown.

Temperament (defined in a multitude of ways but usually encompassing domains related to dispositions in affect, attention, and sociability) has long been examined as an identifiable risk factor for specific psychopathologies.^2^ The concept of temperament in obsessive-compulsive and related disorders (a general category of disorders that currently encompasses TTM and SPD) has been only sparsely examined. Studies using the Tridimensional Personality Questionnaire (TPQ) in adults with obsessive-compulsive disorder (OCD) have found associations between OCD and harm avoidance (characterized by anticipatory worry, fear of uncertainty, shyness, and fatigability).^3,4^

Kim et al^5^ compared personality dimensions in 33 patients with gambling disorder to those of 41 patients with OCD; 40 controls were included. Patients with OCD scored high on harm avoidance (particularly the subdomains of fear of uncertainty and anticipatory worry), while those with gambling disorder demonstrated elevated scores on impulsiveness, extravagance, and novelty seeking (ie, a predisposition towards novel and exploratory behaviors).^6,7^ Lochner et al^8^ used the Temperament and Character Inventory (a newer version of the TPQ) to assess 54 participants with TTM and 278 with OCD. They found that those with TTM reported significantly higher novelty seeking, whereas those with OCD scored significantly greater in harm avoidance. In another study of 21 patients with SPD, Lochner et al^9^ used the TPQ and found that compared to published normative data, individuals with SPD scored higher on measures of reward dependence (ie, tendency to be sentimental, persistent, dependent, and readily form attachments with others) and harm avoidance, but not on novelty seeking.^9^

This limited, and perhaps mixed, literature on temperament in TTM and SPD leaves gaps in our knowledge. It is unclear whether people with TTM and SPD are more harm avoidant, as in OCD, or at least in the case of TTM are more novelty seeking, such as seen in gambling disorder.

Reports on temperament in TTM and SPD are few and neither of the previous 2 studies used their own controls for comparison. (One used OCD as a comparison group and the other used published normative data.) This study used the TPQ in a well-characterized sample of adults with either TTM or SPD to identify temperament and its possible association with symptom severity. Based on prior literature, we hypothesized that people with TTM or SPD would score higher than controls on measures of harm avoidance and reward dependence.

## Methods

### Participants

Non–treatment seeking adults with who met DSM-5 criteria for TTM (n = 44) or SPD (n = 30), and 128 controls were enrolled in a study examining temperament. Inclusion criteria were age 18 to 65 and a primary psychiatric disorder of TTM or SPD. (Those with both TTM and SPD were asked which disorder was the primary problem in terms of daily symptoms and interference.) Exclusion criteria included the inability to provide consent. Nonaffected controls were excluded if they had any current or lifetime psychiatric disorder.

After receiving a thorough explanation of study procedures and an opportunity to ask any questions, participants provided written informed consent. The authors assert that all procedures contributing to this work comply with the ethical standards of the relevant national and institutional committees on human experimentation and with the Helsinki Declaration of 1975, as revised in 2008. All procedures involving human subjects/patients were approved by the University of Chicago institutional review board.

### Assessments

Participants completed a semistructured interview from which information on demographics and clinical characteristics of TTM and SPD was acquired; they also completed the Mini-International Neuropsychiatric Interview.^10^ Additionally, all participants completed the TPQ,^11^ the Massachusetts General Hospital Hairpulling Scale (MGH-HPS)^12^ to assess current severity of TTM symptoms, the MGH-HPS modified for skin picking to characterize severity of SPD symptoms, and the Quality of Life Inventory (QOLI).^13^

#### Tridimensional Personality Questionnaire

The TPQ is a 100-question true-false self-report measure that seeks to measure 3 dimensions (traits) of temperament:

- novelty seeking: exploratory excitability, impulsiveness, extravagance, disorderliness
- harm avoidance: anticipatory worry, fear of uncertainty, shyness with strangers, fatigability
- reward dependence: sentimentality, persistence, attachment, dependence.

Each dimension has 4 subscales.

#### Massachusetts General Hospital Hairpulling Scale

The MGH-HPS is a 7-question self-report scale targeting the individual’s urge to pull, actual pulling, and the consequences of pulling. Each item is scored on a 5-point scale from 0 = no symptoms to 4 = severe symptoms. The item scores are summed to produce a total score (range 0 to 28). This scale has been modified for SPD (MGH-SPD), with a similar 7-item self-report of urges, picking, and consequences of picking.

#### Quality of Life Inventory

The QOLI, an empirically validated, self-report scale consisting of 16 domains of life: love, work, children, health, self-esteem, goals and values, money, play, learning, creativity, helping, friends, relatives, home, neighborhood, and community. The composite scores of all domains provide a measurement of perceived QOL. Very low (T-Score range: 0 to 36) and low (T-Score range: 37 to 42) scores indicate general unhappiness and lower QOL, whereas average (T-Score range: 43 to 57) to high scores (T-Score range: 58 to 77) are considered to indicate lower levels of psychological distress and higher QOL.

### Statistical analysis

Comparisons of TPQ scores between groups were analyzed with *t* tests. Statistical significance was associated with a *P* value <.05. For any significant TPQ total domain measures in those with TTM or SPD, we also explored relationships with symptom severity and quality of life using Spearman’s rho (*R*).

## Results

The sample comprised 202 individuals; 44 had TTM, 30 had SPD, and 128 were controls. The demographics of the sample are presented in TABLE 1.

TPQ scores for the TTM and SPD groups and controls are shown in TABLE 2. Those with TTM or SPD reported significantly elevated harm avoidance scores compared to controls, with TTM scoring significantly higher than SPD. Those with TTM or SPD scored significantly higher than controls on every subscale of harm avoidance: anticipatory worry, fear of uncertainty, shyness with strangers, and fatigability (all *P* < .0001). Those with TTM or SPD also scored significantly higher than controls on 1 subscale of novelty seeking (ie, the subscale of extravagance) (*P* = .0034).

In people with TTM or SPD, higher TPQ total harm avoidance correlated significantly with worse severity of hair pulling (MGH-HPS, *R* = 0.276, *P* =.0496) but not with worse picking (MGH-SPD, *R* = -0.172, *P* = .217). Higher harm avoidance total scores in those with TTM or SPD were also significantly correlated with worse quality of life (*R* = -0.423, *P* = .0002).

## Discussion

To our knowledge, this is 1 of the few studies to examine temperament in adults with either TTM or SPD. We also included a control group to contextualize the findings. The main purpose of our study was the identification of different patterns of personality dimensions in a sample of adults with TTM or SPD. In particular, participants with TTM or SPD mainly showed higher TPQ scores in harm avoidance, along with elevated scores on 1 subdomain of novelty seeking (extravagance).

Higher levels of harm avoidance were expected in this population. Harm avoidance is a temperamental disposition associated with anticipatory worry and fear of uncertainty. This may account for the elevated rates of co-occurring anxiety disorders seen in people with TTM or SPD^14^ and may further support the idea that hair pulling and skin picking may constitute, at least in some people, a self-soothing mechanism.^15^ Elevated harm avoidance scores have been consistently seen in individuals with OCD.^3,6^ Elevated avoidance scores (reflecting a general neuroticism) could be seen as a nonspecific index of anxiety disorders^16^ or may reflect subclinical depressive symptoms (as it is susceptible to mood states), and therefore may only indirectly relate to pulling or picking behavior.

What should be made of the 1 subdomain of novelty seeking, the trait of extravagance (eg, “I often spend money until I run out of cash or get into debt”), being higher in TTM and SPD? Although TTM and SPD have been grouped with OCD as part of an obsessive-compulsive spectrum, these disorders (at least TTM) were originally considered to be part of an impulsive spectrum in DSM-IV. There may be a unique impulsive aspect to both TTM and SPD^17^ that could, in turn, explain the lack of a clear treatment approach to these disorders. This may suggest that treatment approaches that focus on harm avoidance but that also have some focus on impulsivity may be potentially useful.

These results also differ somewhat from the limited previous research. In 2 separate studies, Lochner et al^8,9^ found that TTM was associated with elevated novelty seeking scores and SPD was associated with elevated reward dependence scores. Neither finding was supported by our data except the 1 subdomain of novelty seeking (extravagance) that was elevated. The reasons for these differences could be that one of Lochner et al’s studies used a slightly different version of the TPQ and neither study included internal control data. (One compared against OCD rather than controls; the other compared against external control norms.)

### Limitations

This study has several limitations. Although the sample size had adequate power for the comparisons, the sample size is still relatively small. Because TTM and SPD often have both compulsive and impulsive features, a small sample may not adequately reflect the complexity of these disorders. Furthermore, this study was neither designed nor powered to address whether particular comorbidities contribute to the personality differences identified. This would be interesting to study but would necessitate a much larger sample size.

## Conclusions

Our study has identified some differences in temperament among adults with TTM, SPD, and controls. As such, these findings add to the debate surrounding our understanding of these disorders. These findings are, of course, preliminary. Further research using a dimensional approach to personality may be needed to elucidate the full complexity of TTM and SPD.

## Figures and Tables

**Table 1:** Demographic and clinical characteristics of the 202 participants

Assessment	Trichotillomania (n = 44)	Skin picking disorder (n = 30)	Nonaffected controls (n = 128)
Age, y, mean (SD)	29.95 (8.97)	33.67 (11.72)	21.5 (3.37)
Gender, female, n (%)	36 (81.8)	22 (73.3)	52 (40.6)
Education, n, (%)			
Less than high school	1 (2.3)	0 (0)	2 (1.5)
High school graduate	4 (9.1)	1 (3.3)	7 (5.5)
Some college	10 (22.7)	5 (16.7)	79 (61.7)
College graduate or more	29 (65.9)	24 (80.0)	40 (31.3)
Race, n (%)			
White	33 (75.0)	26 (86.67)	113 (88.3)
Other	11 (25.0)	4 (13.33)	15 (11.7)
Any current comorbidity, n (%)	19 (43.2)	21 (70.0)	0 (0)
MGH-HPS or MGH-SPD score	17.4 (SD = 5.4)	16.6 (SD = 5.3)	-
Quality of Life Inventory score	78.59	89.12	111.57

MGH-HPS: Massachusetts General Hospital Hairpulling Scale; MGH-SPD: Massachusetts General Hospital Hairpulling Scale Modified for Skin Picking.

**Table 2:** Temperament in trichotillomania and skin picking disorder vs healthy controls

TPQ scores	TTM (n = 44)	SPD (n = 30)	HC (n = 128)	Kruskal-Wallis/Wilcoxon	*P*	Difference between groups
Novelty seeking	14.77	16.93	16.03	3.056	.217	-
Exploratory excitability	4.39	5.07	4.92	2.72	.256	-
Impulsiveness	2.23	2.73	2.81	2.58	.275	-
Extravagance	3.84	4.03	2.99	11.40	.0034	TTM & SPD > HC
Disorderliness	4.32	5.10	5.31	6.499	.0388	-
Harm avoidance	19.77	16.27	10.92	43.768	<.0001	TTM & SPD > HC TTM > SPD
Anticipatory worry	5.36	4.53	3.27	23.232	<.0001	TTM & SPD > HC
Fear of uncertainty	4.02	3.20	2.18	25.953	<.0001	TTM & SPD > HC
Shyness with strangers	4.64	3.87	2.64	24.884	<.0001	TTM & SPD > HC
Fatigability	5.75	4.67	2.83	38.198	<.0001	TTM & SPD > HC
Reward dependence	17.48	15.93	16.98	1.6449	.439	-
Sentimentality	3.36	3.37	3.24	0.7107	.701	-
Persistence	5.43	4.73	4.79	2.8134	.245	-
Attachment	6.11	5.43	6.17	1.4456	.485	-
Dependence	2.57	2.40	2.80	2.994	.224	-

HC: healthy controls; SPD: skin picking disorder; TTM: trichotillomania; TPQ: Tridimensional Personality Questionnaire.
